# Continuous Patient Monitoring in the Catheterization Laboratory: Usability and Operational Impact Assessment

**DOI:** 10.7759/cureus.105978

**Published:** 2026-03-27

**Authors:** Raul E Herrera, Kevin J Nicholas, Nadia Edelman, Eddy Napoles, Lordany Milanes, Tashima Lindo, Melinda Caraballo, Jochen Weichert, Alexander Haak, Britt van Rooij, Marcus E St John

**Affiliations:** 1 Research and Outcomes, Miami Cardiac and Vascular Institute, Baptist Health South Florida, Miami, USA; 2 Clinical Operations, Miami Cardiac and Vascular Institute, Baptist Health South Florida, Miami, USA; 3 Patient Care, Invasive Services, Miami Cardiac and Vascular Institute, Baptist Health South Florida, Miami, USA; 4 Cardiac Cath Lab, Miami Cardiac and Vascular Institute, Baptist Health South Florida, Miami, USA; 5 Patient Care, Cardiac Cath Lab, Miami Cardiac and Vascular Institute, Baptist Health South Florida, Miami, USA; 6 Clinical Development, Philips Netherlands BV, Eindhoven, NLD; 7 Clinical Sciences, Philips North America LLC, Miami, USA; 8 Clinical Sciences, Philips Medical Systems Best BV, Eindhoven, NLD

**Keywords:** catheterization laboratory, cath-lab efficiency, device integration, patient monitoring, preparation time, usability

## Abstract

Background

Currently, there exists no evidence on how workflows impact staff and work efficiency when patient monitors travel with patients and integrate into the catheterization laboratory (cath-lab). This prospective, pre/post study compares a Continuous Patient Monitoring (CPM) workflow to a workflow switching between stationary patient monitors (stationary) in a cath-lab environment.

Methods

The Philips Hemodynamic Monitoring System with IntelliVue X3, which can work with the monitoring devices (MX700 with IntelliVue X3) from the holding area (six bays), was installed in two cath-labs. Retrospectively, timestamps were collected by the staff before the introduction of CPM. After a four-week training and washout period, the staff collected timestamps for the CPM-phase and answered a survey containing context- and usability-specific questions (Usefulness, Satisfaction, and Ease of Use (USE) and System Usability Scale (SUS) questionnaires).

Results

The CPM workflow was implemented on the 6th of March 2023, and 15 cath-lab nurses participated in the CPM usability survey. The survey resulted in an SUS score of 70% (N = 15) and a USE score of, on average, 6 out of 7 (N = 15), with ease of learning (N = 15, mean: 6.7 ± 0.57) and ease of use (N = 15, mean: 6.4 ± 0.66). Qualitative feedback indicated that the CPM workflow may save time, is self-explanatory, is easy to use, and results in consistent monitoring of the patients' vital signs. For operational metrics, a total of 1042 patients were included (stationary phase: 444, CPM phase: 598) in this study. The difference in means between the pre-preparation times for the stationary phase (N = 438, 12.8 ± 4.95 min) and the CPM phase (N = 433, 13.1 ± 5.58 min) is -0.32 (-1.02, 0.38) minutes. The Hodges-Lehmann difference in medians is 0.15 (-0.52, 0.80) minutes. For the post-preparation times, the difference in means between the stationary phase (N = 416, 10.6 ± 4.27 min) and the CPM phase (N = 412, 11.3 ± 5.47 min) is -0.65 (-1.31, 0.03) minutes. The Hodges-Lehmann difference in medians is 0.32 (-0.23, 0.87) minutes.

Conclusion

CPM has been successfully implemented in the cath-lab environment. SUS was above the published average values, and the USE indicated that the ease of learning and ease of use scored high for the system. The USE score results were not significantly different from USE scores for CPM in the perioperative setting. No clinically meaningful difference in patient preparation times was found. The staff experienced an increased sense of patient safety and were positive about the usability of CPM.

## Introduction

Healthcare organizations face the challenge of treating more patients to become more cost-effective while ensuring the safety and quality of care, and enhancing staff experience and patient satisfaction. The cardiac catheterization laboratory (cath-lab) environment is especially susceptible to inefficiencies due to growing patient populations, staff shortage, and new minimally invasive procedures, which add complexity to an overloaded workforce [1-3]. Cath-lab utilization may experience high turnover time and become a bottleneck in the treatment of patients. Research focused on efficiency in the cath-lab is, however, rather limited. In the literature, a few studies analyzed cath-lab processes using well-known management methodologies (e.g., Lean, sigma six). These methodologies focus on identifying and eliminating redundant non-value adding activities [4,5]. Agarwal et al. reported that eliminating redundancies and streamlining workflow improved operational efficiency in the cath-lab [4]. The study looked at cath-lab turnaround time (i.e., inter-procedural turnover time) and physician downtime. A study performed by Reed et al. also set out to improve cath-lab efficiency [5] by systematically reducing operational inefficiencies in the cath-lab. Through a stepwise implementation of specific changes, the group improved cath-lab start times, reduced turnaround times, and increased overall productivity. This increasing workload in the cath-lab results in staff burnout and can contribute to poorer patient satisfaction. 

Philips introduced a continuous patient monitoring (CPM) workflow for the cath-lab. The CPM workflow makes use of the IntelliVue X3, a transport monitor, which is integrated in the monitoring devices present in the holding area and the monitoring system in the cath-lab. The IntelliVue X3 travels with the patients, from the holding area to the cath-lab and back to the holding area. The workflow makes it possible to collect patient data during the patient journey in the hospital. CPM could potentially improve safety and quality of care. Another advantage of the CPM workflow is that the patient is always monitored, e.g., at times the patient needs to wait in the cath-lab for an available bed in the post-holding area, the patient will be monitored. Patient preparation activities related to attaching the patient to the hemo system in the cath-lab will be reduced and can result in a more efficient use of the cath-lab. This study focuses on the CPM workflow in the cath-lab; however, the workflow has already shown its potential in the OR environment [6]. 

The primary objective of the study was to capture the staff experience after changing from a stationary workflow to the CPM workflow [7] using a survey and anecdotal feedback. The secondary objective was to investigate the potential change in time metrics with a focus on patient preparation times pre- and post-procedure in the cath-lab. Patient preparation time may change by shifting activities into the pre- or post-holding area or by an improvement in efficiency. Examples of these activities are preparing the chest, placing the ECG patches and leads, and entering patient demographics into the monitoring system. An exploratory objective was to document the staff-perceived patient comfort based on a survey and anecdotal feedback.

## Materials and methods

The study was performed at Miami Cardiac and Vascular Institute (MCVI) of Baptist Health South Florida, Miami, FL. Designated a Magnet hospital, the MCVI carries out thousands of cardiac and vascular surgeries and about 30,000 interventional procedures per year in the fields of cardiology, interventional radiology, cardiac electrophysiology, interventional cardiology, structural heart, and interventional neuroradiology. MCVI's Institutional Review Board approved the study (1846532) and waived the need for informed consent from patients, while informed consent from staff was obtained. The inclusion criteria for the cath-lab nurses and technicians were age ≥18 years and that they utilized the new CPM workflow. Exclusion criteria for the study were emergency patients, patients undergoing a structural heart procedure, patients under anesthesia, and patients who did not enter the cath-lab via the holding area. 

The study included six beds in the pre-holding area and two cath-labs. For the stationary workflow, patients were monitored in the pre-holding area using IntelliVue MX700 (Philips, Böblingen, Germany) patient monitors equipped with MMS measurement units and the cath-labs with the Xper Flex Cardio Physio Monitoring System (Philips, Florida, United States). The CPM workflow introduced eight (six operational, two spares) IntelliVue X3 transport monitors (Philips, Böblingen, Germany) to the pre-holding area and upgraded the cath-lab hemodynamic system to the Philips Hemo System with IntelliVue X3 (Philips, Best, The Netherlands), with each one spare X3. 

In the stationary workflow, the patients were monitored in the pre-holding area. Next to monitoring the patient, other preparation activities were performed in the pre-holding area, such as shaving the chest for electrode placement. When the patient was picked up and transported to the cath-lab, the monitoring of the patient stopped. In the cath-lab, the patient was prepared for monitoring during the procedure by the attachment of radiolucent electrodes, the non-invasive blood pressure (NIBP) cuff, and the SpO_2_ sensor to the patient. After the procedure, the patient's cables and ECG patches were removed, and the unmonitored patient was transported to the post-holding area. 

**Figure 1:**
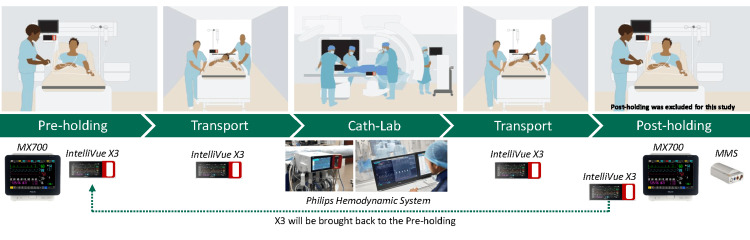
Continuous Patient Monitoring (CPM) workflow: schematic overview of the CPM workflow. Figure created by the authors with MS Powerpoint (Microsoft 365, version 2602, Microsoft Corp, Redmond, WA, USA)

A depiction of the CPM workflow is shown in Figure 1. During the CPM workflow, the patient in the pre-holding area was prepared for the procedure in the cath-lab. To make uninterrupted monitoring possible, the positioning of the ECG electrodes has been adapted to the cath-lab's preferred setup. In addition, radiolucent ECG patches were introduced in the six bays in the pre-holding area. All sensor cables (ECG, SpO_2_, and NIBP) were attached to the IntelliVue X3 in the pre-holding area. The X3 was undocked from the MX700 monitoring unit while cables remained attached, monitoring the patient during transport. In the cath-lab, the IntelliVue X3 docked into the Philips Hemo System. After the procedure, the IntelliVue X3 was undocked and travelled with the patient to the post-holding area. Here, the patient cables were attached to the monitoring system in the post-holding. The X3 was brought back to the pre-holding area to make sure the IntelliVue X3 is available for the next patient. Time metrics used in the study were collected in the cath-lab data management solution XperIM (Philips, Best, The Netherlands).

The CPM workflow was evaluated quantitatively and qualitatively via a questionnaire to gain insights into staff and patient experience. The target audience of the questionnaire was the cath-lab nurses and technicians. MCVI research coordinators obtained consent and collected filled-out surveys, resulting in a convenience sample. Nurses filled out the survey in multiple sessions during the shift. These sessions took place over the span of three weeks. The patient’s experience was indirectly measured via the staff using three specific statements (five-point scale) on patient comfort regarding the usage of the Philips Hemo System with the IntelliVue X3 (see Table 1). No patients were surveyed during this study. The staff satisfaction was measured using the System Usability Scale (SUS) questions, Net Promoter Score (NPS), and Usefulness, Satisfaction, and Ease of Use (USE) assessment questions, which are standardized, non-proprietary instruments made freely available for research and industrial purposes. The USE score measures the subjective usability of a product or service [8]. It is a 30-item survey that examines four dimensions of usability: usefulness, ease of use, ease of learning, and satisfaction. The overall score correlates very well with the results of the SUS [9]. Although the SUS is better validated, the USE score allows us to measure the properties simultaneously. The NPS assesses the willingness of the staff to recommend the CPM workflow to other people. It is calculated by subtracting the percentages of the detractors and the promoters, where promoters scored 9 or 10, and detractors scored below 7. The qualitative part of the questionnaire consisted of open questions assessing the staff's experience with the workflow change. The patient's satisfaction was measured indirectly, asking the staff's opinion using a Likert scale questionnaire (Tables 1, Appendix). Qualitative endpoints were considered exploratory endpoints, and the minimum sample size of the convenience sample was chosen to be 15 in accordance with the FDA usability guidance document [10]. All questionnaires used can be found in the appendix of this paper.

**Table 1:** Patient’s experience statements that were part of the questionnaire.

Statement
The overall patient comfort is improved by using the Philips Hemo System with the IntelliVue X3.
Philips Hemo System with the IntelliVue X3 improves patient satisfaction and comfort due to less preparation time on the narrow cath lab table in an intimidating, sterile environment.
Patient comfort is improved by a reduced amount of re-cabling needed with the Philips Hemo System with the IntelliVue X3, that leads to fewer disturbing interactions with the patient.

The CPM workflow efficiency was evaluated using time metric data regarding the patient preparation times before (stationary data set) and after the continuous patient monitoring (CPM data set) workflow was introduced. The time metric data was manually entered by the cath-lab staff into XperIM. All time metrics used in the study had been collected routinely by MCVI prior to the study to get insights into their cath-lab efficiency. At the end of each phase, the time metric data was extracted from the Xper Information Management system. From this time metric data, two types of preparation times in the cath-lab were obtained. The pre-preparation time, which is defined as the time between the patient's entering the cath-lab and the MD's arrival, and the post-preparation time, which is the time between the end of the procedure and the moment the patient leaves the cath-lab. 

To investigate whether the introduction of the CPM workflow will reduce the patient preparation time, statistical testing was performed on the time metric data. Distributions are tested for normality using the Shapiro-Wilk test. We report differences in means with Welch-Satterthwaite confidence intervals and Hodges-Lehmann differences in median. These metrics are reported for the complete data set without outliers removed and for a data set with outliers defined as longer than twice the mean, 25 minutes in the pre-preparation time, and 20 minutes in the post-preparation time. The usability scores were compared to usability scores from the perioperative setting using the Wilcoxon rank-sum test. Statistical significance was determined as p < 0.05. 

## Results

**Figure 2:**
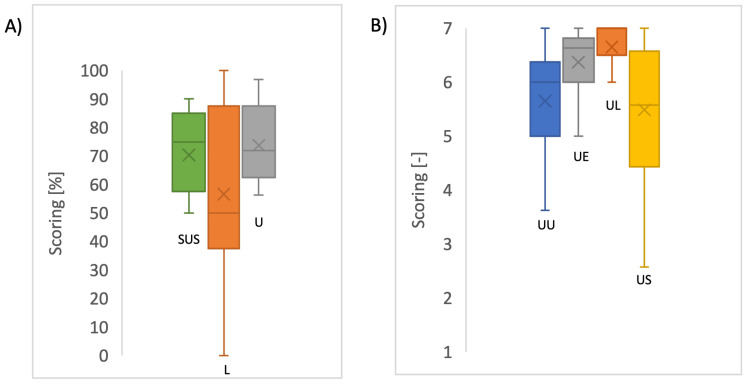
(A) The System Usability Scale (SUS) and (B) the Usefulness, Satisfaction, and Ease of Use (USE) assessment. L = Learnability, U = Usability, UU = Usefulness, UE = Ease of Use, UL = Ease of Learning, US = Satisfaction The data are represented as a box plot, indicating the median as a line in the box, the mean as X, the box represents the 25% to 75% percentile, and the whiskers represent the minimum and maximum. Image created by the authors with Microsoft Excel (Microsoft 365, version 2602, Microsoft Corp, Redmond, WA, USA)

The primary objective of this study is to collect staff and patient experiences. A survey was conducted to collect feedback from 15 cath-lab nurses and technicians. The SUS for CPM workflow in the cath-lab environment is 70% (N = 15). In addition, the USE score resulted in an average of 6 (N = 15) on a scale of 1 (strongly disagree) to 7 (strongly agree). Figure 2 shows the SUS and USE scores in detail. As shown in Figure 2, the ease of use and ease of learning of the CPM workflow score high as part of the USE score. In the USE score, the largest variability is found in the Satisfaction part of the questionnaire. The SUS can be divided into two categories: learnability and usability. Figure 2 shows that the learnability has a large variability, which is contradictory to the ease of learning score in the USE assessment. 

The patient experience is measured using the statements given in Table 1. These statements are rated high with an average of 4 out of 5. The NPS, which asks the staff how likely they are to recommend the system (CPM workflow) to a friend or colleague, is rated with a score of 31% (N = 13). This means that the CPM workflow has more promoters than detractors.

In the open questions, the participants stated that the CPM workflow can save time; it is very self-explanatory, simple to apply, and results in consistent monitoring of the patient’s vital signs. Cable management and having the solution in the post-holding area were marked as “improvements." Next to the survey outcome, direct feedback from the staff indicated that the patient safety advantage provided by the CPM workflow contributed to peace of mind and a positive experience for patients and caregivers. In addition, the staff stated that patient experience is improved due to reduced ECG electrode repatching in the holding area and cath-lab. 

The second objective of this study is to investigate the potential reduction of patient preparation times in the cath-lab, pre- and post-procedure. For this objective, time metric data was studied that was recorded by the staff as part of their regular work. In total, 444 procedures for the stationary data set and 598 procedures for the CPM dataset matched the study criteria and were included, see Table 2. Some procedures have been excluded from the data set for the pre- or post-preparation time. The time intervals were filtered on the following criteria: when the start preparation time was the same as the start of the procedure, when the start preparation time or the start of the procedure time was not filled out, or when the preparation time was less than one minute.

**Table 2:** Number of procedures for the stationary and Continuous Patient Monitoring (CPM) dataset used for the pre-preparation time. The data are represented as % (N).

	Stationary dataset	CPM dataset
# procedures	100% (N = 444)	100% (N = 598)
Room 1	39% (N = 171)	38% (N = 229)
Room 2	61% (N = 273)	62% (N = 369)
Procedure types		
Diagnostic	75% (N = 331)	75% (N = 450)
Cath and intervention	20% (N = 88)	18% (N = 108)
Coronary intervention	5.6% (N = 25)	6.7% (N = 40)

**Figure 3:**
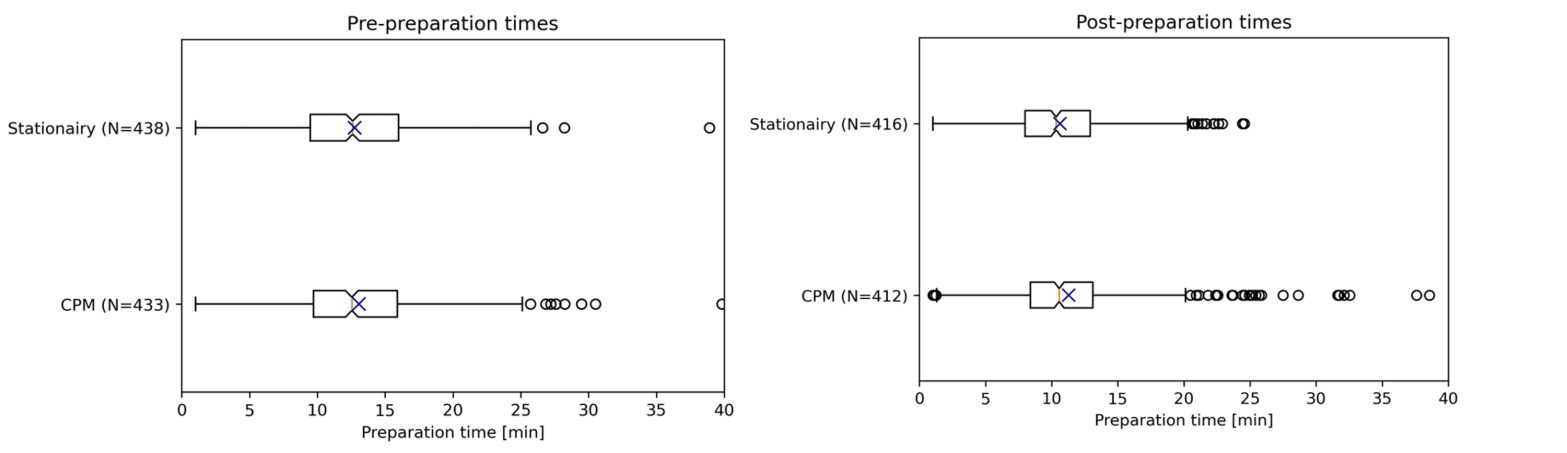
The pre- (left) and post-preparation (right) times for the stationary and Continuous Patient Monitoring (CPM)-phase derived from the time metrics. The data are represented as box-plot indicating N next to the label. In the plot, the mean is represented as X, the line as the median, and the box as the 25% to 75% percentile, 1.5 times the interquartile range as whiskers and outliers as circles. Image created by the authors with Python 3.12, matplotlib (Python Software Foundation, version 3.12, 2023, www.python.org/doc/)

The pre-preparation and post-preparation derived from the time metric data are shown in Figure 3. For the data including the outliers the Shapiro-Wilk test indicates the data is not normal for all four distributions, stationary pre-preparation time (statistic = 0.9834, p-value = 0.0001, skewness = 0.52, kurtosis = 1.37), CPM pre-preparation time (statistic = 0.9039, p-value = 0.0, skewness = 1.85, kurtosis = 11.85), stationary post-preparation time (statistic=0.9800, p-value=0.0, skewness = 0.44, kurtosis= 0.611), and CPM post-preparation time (statistic = 0.8959, p-value = 0.0, skewness = 1.45, kurtosis = 4.30). The difference in means between the pre-preparation times for the stationary phase (N = 438, 12.8 ± 4.95 minutes) and the CPM phase (N = 433, 13.1 ± 5.58 minutes) is -0.32 minutes with a 95% confidence interval of (-1.02, 0.38) minutes. The Hodges-Lehmann difference in medians is 0.15 (-0.52, 0.80) minutes. For the post-preparation times, the difference in means between the stationary phase (N = 416, 10.6 ± 4.27 minutes) and the CPM phase (N = 412, 11.3 ± 5.47 minutes) is -0.65 minutes with a 95% confidence interval of (-1.31, 0.03) minutes. The Hodges-Lehmann difference in medians is 0.32 (-0.23, 0.87) minutes.

In the dataset with outliers removed, the Shapiro-Wilk test indicates that the data are normal for the stationary pre-preparation time (statistic = 0.9959, p-value = 0.3308, skewness = 0.07, kurtosis = -0.37), normal for the CPM pre-preparation time (statistic = 0.9966, p-value = 0.5105, skewness = 0.11, kurtosis = -0.08), not normal for the stationary post-preparation time statistic = 0.9914, p-value = 0.0197, skewness = -0.04, kurtosis = -0.04), and not normal for the post-preparation time (statistic = 0.9838, p-value = 0.0003, skewness= -0.17, kurtosis = 0.22). The difference in means between the pre-preparation times between the stationary phase (N = 432, 12.5 ± 4.59 minutes) and the CPM phase (N = 422, 12.6 ± 4.53 minutes) is -0.05 minutes with a 95% confidence interval of (-0.67, 0.56) minutes. The Hodges-Lehmann difference in medians is 0.05 (-0.58, 0.68) minutes. For the post-preparation times, the difference in means between the stationary phase (N = 401, 10.2 ± 3.74 minutes) and the CPM phase (N = 384, 10.2 ± 3.73 minutes) is -0.01 minutes with a 95% confidence interval of (-0.54, 0.51) minutes. The Hodges-Lehmann difference in medians is 0.08 (-0.42, 0.60) minutes.

## Discussion

To the best of the authors' knowledge, the present study is the only known study introducing new medical device technology changing the workflow to impact cath-lab efficiency, staff, and, indirectly, patient experience. The CPM workflow was implemented in two cath-labs and six holding beds at MCVI, Baptist Health South Florida, Miami, FL. The study assesses staff and patient experience using a survey. In addition, efficiency improvement in the cath-lab is studied by comparing the cath-lab preparation times prior to and post-CPM implementation. We showed that the CPM workflow was easy to learn and easy to use from the usability scores. The indirectly measured patient experiences scored high, and this was also underlined by anecdotal reporting through the staff. This anecdotal reporting shows that CPM improves patient safety and staff’s peace of mind by monitoring the patient without interruption. The captured time metric data shows that the patient preparation time did not change significantly for the CPM workflow.

From the questionnaire, an SUS of 70% (N = 15) is considered acceptable and above average [11]. It is remarkable that the learnability of the CPM workflow has been scored differently in the SUS and USE scores. The ease of learning scored very high in the USE assessment and much lower in the SUS. A potential reason for this lower score and high variability could be the setup of the SUS questions. The learnability questions are inverted statements. A participant could have missed the inversion of the questions, which has a negative influence on the scoring. In the open questions of the survey, staff members mentioned that the CPM workflow is self-explanatory and simple to apply. This is in line with the high score in the learnability and ease of use of the USE assessment.

The continuous patient monitoring workflow has been studied as well by Tourelle et al. [6] for intrahospital transfer of perioperative patients. They showed that the staff were more satisfied in terms of flexibility, cleanability, and usability using the transport monitor IntelliVue X3. In the perioperative setting usability from the USE questionnaire was not significantly different (median (25%; 75%): perioperative 5.1 (4.68; 5.25), CPM 6 (5.13; 6.25), p = 0.06), easy of use (median (25%; 75%]: perioperative 5.1 (4.05; 5.66), CPM 6 (6.14; 6,77), p < 0.0001) and ease of learning (median (25%; 75%): perioperative 5.6 (4.19; 6.19), CPM 7 (6.63; 7), p < 0.001) were significantly different, while satisfaction (median (25%; 75%): perioperative 5.4 (4.04; 5.71), CPM 5.6 (4.64; 6.5), p = 0.159) was not significantly different. Increases in ease of use and ease of learning might be due to the fact that MCVI already used the predecessor Philips products, while Tourelle et al. [6] used a competitor system before.

Other qualitative feedback was based on the consistent monitoring of the patient’s vital signs, which may increase the safety of the patient. One staff member reported improved safety for a patient experiencing a code. The patient needed to be sent to the CT and X-ray. Because the patient was always monitored by the IntelliVue X3, they did not need to connect the patient to a transport monitor and were able to immediately proceed with transporting the patient, saving time. This example shows a benefit CPM may have on patient safety.

Another aspect mentioned in the anecdotal feedback was related to enhanced sustainability: a possible reduction in ECG electrode waste. The CPM workflow encourages the usage of the same translucent ECG electrode patches in the pre-holding area, which was accomplished after training nursing staff on the usage and positioning of the radiolucent electrodes. Additionally, an increased patient satisfaction was reported by the staff related to avoiding several electrode removals and placements. This is an especially relevant benefit for the elderly patients who often have very thin skin, which is vulnerable to injury when removing electrodes.

This study showed that the pre- and post-preparation times were not significantly different for the stationary and CPM workflow. A reason could be that MCVI had already highly optimized its workflow. They moved several of their preparation activities from the cath-lab to the pre-holding area to reduce the preparation time in the cath-lab. Tasks such as preparation of the chest and groin area and wrist preparation have been performed in the pre-holding before the introduction of CPM. The extra activities that are moved from the cath-lab to the pre-holding area during this study are the placement of the radiolucent electrodes and attachment of ECG cables, and the attachment of the SpO2 and NIBP measurements, which may only marginally improve preparation time. Cable management could be a reason that no improvement in preparation time was found. This was mentioned in the survey as an improvement. Due to cable management, staff might need to spend the time saved untangling cables or re-attaching ECG leads. In the survey, some of the staff members mentioned that the CPM workflow saves time, even though statistically there was no difference in the preparation time. The discrepancy between the perceived and measured preparation time might be due to a reduction of the total number of steps the staff experience, while performing individual steps may take longer. This perception might positively influence the perceived work pressure of the staff members. 

Another discussion point of the time metric data is data quality. The staff entered timestamps manually into XperIM during routine care, which may be prone to human errors. In the results section, filtering of the data to reduce the amount of faulty entered data is explained. Future studies might improve data quality by employing a real-time location system using RFID tags to minimize manual entry. Though there are many articles on use cases for real-time location systems [12], there is no article comparing the accuracy of manual entry of process times to process times captured with real-time location systems. Stankiewicz et al. [13] found that RFID badge swiping improved the compliance with data entry.

Literature about time metrics and efficiency in the cath-lab is limited and difficult to compare with the preparation times of this study. As discussed in the introduction, Reed et al. [1,5] showed that the average turnover times are 16.4 minutes. This is the time between two procedures, which was not measured in this study. Van der Graaf et al. [14] investigated workflow and efficiency optimizations in the cath-lab. A pre-preparation time in the cath-lab of 11.8 ± 3.8 minutes was reported, which is in the same range reported in this study (stationary: 12.8 ± 4.95 minutes, CPM: 13.1 ± 5.58 minutes). A similar preparation time was reported with an average of 20.42 ± 9.9 minutes in [15] for a Dutch cath-lab. This paper found that the preparation times were not dependent on patient characteristics such as age, BMI, and gender. In addition, Istaphanous et al. [2] studied the causes of delay in a catheterization laboratory and reported 15.1 minutes as the time to surgical incision. In the latter study, Six Sigma principles were used to improve the time to start the surgery. Tourelle et al. [6] in their study on intrahospital transfer of perioperative patients showed that time was saved during the patient handover between departments.

We showed that the usability of the uninterrupted monitoring workflow in the cath-lab is good. In the survey, cable management was called out as an area to improve usability further. During the study, the cable management was mitigated by sliding the patient over from the bed to the table. This reduced the risk of entangled cables and detached ECG leads. Cable management might be addressed in the future through wearables.

A limitation of this study was not having this CPM solution in the post-holding area. This was also mentioned in the questionnaire. Due to not having the solution in the post-holding, the patient cables had to be disconnected from one monitor and connected to the other. This reduced the efficiency gain in the holding. Having the IntelliVue X3 in the post-holding might save time for the cath-lab nurses, so they can pick up the next patient earlier and therefore reduce the patient turnover time. Future studies should include both pre-and post-holding areas into account.

A second limitation of the study is that the survey was only conducted after the implementation of the CPM workflow and with a limited sample. It would have been valuable to compare the stationary and CPM workflow in the same hospital. Though chosen in accordance with the FDA guidance, the achieved sample size of 15 clinicians is small, and results may not generalize to other settings. The results from this study may be used to correctly determine sample sizes, which may mandate multi-center approaches due to the larger sample sizes needed. Therefore, future studies replicating the questionnaire may improve the generalization properties [16]. The last limitation that needs to be discussed is the fact that MCVI was already very efficient in the cath-lab. This limited the ability to improve the preparation times in the cath-lab further. Future studies might consider an observational pilot to benchmark the cath-labs efficiency before considering a pivotal study assessing the new workflow.

This study was a before-and-after study. In the future, a randomized trial focusing on emergency patients might attempt to optimize door-to-balloon time using the Continuous Patient Monitoring workflow. In addition, a retrospective cohort study might more rigorously explore the anecdotal evidence on patient safety aspects for in-hospital patients undergoing cath-lab procedures.

## Conclusions

The CPM workflow has been successfully introduced at MCVI. It is perceived as easy to learn and useful to the staff, as exhibited by an overall USE score rating of 6 out of 7 (strongly agree). Compared to the perioperative setting, CPM achieves superior staff experience regarding ease of use and ease of learning. In the qualitative feedback, patient safety is recognized as the most important benefit of CPM. The indirectly measured patient satisfaction scored high by the staff, and anecdotal feedback pointed specifically to patients with thin skin that might benefit from reduced ECG electrode re-patching. For CPM in the cath-lab and holding area, no efficiency gains were found, which might depend on the baseline efficiency of the units. A future study on emergency patients experiencing more re-patching and handover might lead to significant efficiency gains. Future studies should focus on the potential benefits of CPM, patient safety, and staff satisfaction, taking learnings from this study into account to achieve greater generalizability.

## Appendices

The questionnaire included an exclusion criteria section, as shown in Table 3.

**Table 3:** Exclusion criteria for healthcare providers included in study

Exclusion criteria													
If ‚Yes‘, exclude participant from study								Yes			No		
Participant younger than 18 years (yes/no)													

Table 4 shows the USE score questionnaire used in the study

**Table 4:** USE score questionnaire health care providers andsered in study

Usability assessment (USE score)										
Mark the applicable			Strongly disagree (1) to Strongly agree (7)							
1	It helps me be more effective.			1	2	3	4	5	6	7
2	It helps me be more productive.			1	2	3	4	5	6	7
3	It is useful.			1	2	3	4	5	6	7
4	It gives me more control over the activities in my life.			1	2	3	4	5	6	7
5	It makes the things I want to accomplish easier to get done.			1	2	3	4	5	6	7
6	It saves me time when I use it.			1	2	3	4	5	6	7
7	It meets my needs.			1	2	3	4	5	6	7
8	It does everything I would expect it to do.			1	2	3	4	5	6	7
9	It is easy to use.			1	2	3	4	5	6	7
10	It is simple to use.			1	2	3	4	5	6	7
11	It is user friendly.			1	2	3	4	5	6	7
12	It requires the fewest steps possible to accomplish what I want to do with it			1	2	3	4	5	6	7
13	It is flexible			1	2	3	4	5	6	7
14	Using it is effortless			1	2	3	4	5	6	7
15	I can use it without written instructions.			1	2	3	4	5	6	7
16	I don't notice any inconsistencies as I use it.			1	2	3	4	5	6	7
17	Both occasional and regular users would like it.			1	2	3	4	5	6	7
18	I can recover from mistakes quickly and easily.			1	2	3	4	5	6	7
19	I can use it successfully every time.			1	2	3	4	5	6	7
20	I learned to use it quickly.			1	2	3	4	5	6	7
21	I easily remember how to use it.			1	2	3	4	5	6	7
22	It is easy to learn to use it.			1	2	3	4	5	6	7
23	I quickly became skillful with it.			1	2	3	4	5	6	7
24	I am satisfied with it.			1	2	3	4	5	6	7
25	I would recommend it to a friend.			1	2	3	4	5	6	7
26	It is fun to use.			1	2	3	4	5	6	7
27	It works the way I want it to work.			1	2	3	4	5	6	7
28	It is wonderful.			1	2	3	4	5	6	7
29	I feel I need to have it.			1	2	3	4	5	6	7
30	It is pleasant to use.			1	2	3	4	5	6	7

Study participants were asked to answer structured, specific questions on the system used in the study, as shown in Table 5.

**Table 5:** Specific questions on the Philips Hemo System with the IntelliVue X3 answered by the healthcare providers participating in the study

Specific questions on the Philips Hemo System with the IntelliVue X3								
1 = Strongly disagree, 2 = disagree, 3 = neutral, 4 = agree, 5 = strongly agree								
1	The overall patient comfort is improved by using the Philips Hemo System with the IntelliVue X3.			1	2	3	4	5
2	Philips Hemo System with the IntelliVue X3 improves patient satisfaction and comfort due to less preparation time on the narrow cath lab table in an intimidating, sterile environment.			1	2	3	4	5
3	Patient comfort is improved by a reduced amount of re-cabling needed with the Philips Hemo System with the IntelliVue X3, that leads to fewer disturbing interactions with the patient.			1	2	3	4	5

Study participants were asked to answer the questions of the SUS as shown in Table 6.

**Table 6:** System Usability Scale (SUS) questionnaire answered by healthcare providers participating in the study

System Usability Scale (SUS) score								
1	I think that I would like to use this system frequently			1	2	3	4	5
2	I found the system unnecessarily complex			1	2	3	4	5
3	I thought the system was easy to use			1	2	3	4	5
4	I think that I would need the support of a technical person to be able to use the system			1	2	3	4	5
5	I found the various functions in this system were well integrated			1	2	3	4	5
6	I thought there was too much inconsistency in this system			1	2	3	4	5
7	I would imagine that most people would learn to use this system very quickly			1	2	3	4	5
8	I found the system very cumbersome to use			1	2	3	4	5
9	I felt very confident using the system			1	2	3	4	5
10	I needed to learn a lot of think before I could get going with the system			1	2	3	4	5

Net promoter score was assessed as part of the questionnaire, as shown in Table 7.

**Table 7:** Net promoter score question as part of the questionnaire to health care providers participating in the study

Net promoter score													
Mark the applicable			Not at all likely (1) to Extremely likely (10)										
1	How like are you to recommend the system to a friend or colleague			1	2	3	4	5	6	7	8	9	10

The questionnaire concluded with a section for unstructured feedback, as shown in Table 8.

**Table 8:** Unstructured feedback questions answered by participating healthcare professionals

What was missing or disappointing in your experience with CPM?													
What do you like most about CPM?													
What do you like least about CPM?													
How do you benefit from using CPM?													
How does our product meet your needs?													
What is the one thing we could do to make you happier?													
